# The Small and Dynamic Pre-primed Pool at the Release Site; A Useful Concept to Understand Release Probability and Short-Term Synaptic Plasticity?

**DOI:** 10.3389/fnsyn.2019.00007

**Published:** 2019-03-07

**Authors:** Bengt Gustafsson, Rong Ma, Eric Hanse

**Affiliations:** Department of Physiology, Sahlgrenska Academy, University of Gothenburg, Gothenburg, Sweden

**Keywords:** hippocampus, synapse, nanomodule, release probability, plasticity, glutamate, vesicle, release site

## Abstract

Advanced imaging techniques have revealed that synapses contain nanomodules in which pre- and post-synaptic molecules are brought together to form an integrated subsynaptic component for vesicle release and transmitter reception. Based on data from an electrophysiological study of ours in which release from synapses containing a single nanomodule was induced by brief 50 Hz trains using minimal stimulation, and on data from such imaging studies, we present a possible modus operandi of such a nanomodule. We will describe the techniques and tools used to obtain and analyze the electrophysiological data from single CA3–CA1 hippocampal synapses from the neonatal rat brain. This analysis leads to the proposal that a nanomodule, despite containing a number of release locations, operates as a single release site, releasing at most a single vesicle at a time. In this nanomodule there appears to be two separate sets of release locations, one set that is responsible for release in response to the first few action potentials and another set that produces the release thereafter. The data also suggest that vesicles at the first set of release locations are primed by synaptic inactivity lasting seconds, this synaptic inactivity also resulting in a large heterogeneity in the values for vesicle release probability among the synapses. The number of vesicles being primed at this set of release locations prior to the arrival of an action potential is small (0–3) and varies from train to train. Following the first action potential, this heterogeneity in vesicle release probability largely vanishes in a release-independent manner, shaping a variation in paired-pulse plasticity among the synapses. After the first few action potentials release is produced from the second set of release locations, and is given by vesicles that have been recruited after the onset of synaptic activity. This release depends on the number of such release locations and the recruitment to such a location. The initial heterogeneity in vesicle release probability, its disappearance after a single action potential, and variation in the recruitment to the second set of release locations are instrumental in producing the heterogeneity in short-term synaptic plasticity among these synapses, and can be seen as means to create differential dynamics within a synapse population.

## Introduction

Recent work using various imaging techniques has begun to reveal the supramolecular organization of the presynaptic active zone and of its postsynaptic counterpart, the postsynaptic density. There are still uncertainties regarding the exact spatial relationship among structures vital to the release such as the readily releasable vesicles, the vesicle scaffold proteins, the voltage-gated calcium channels (VGCCs), as well as the postsynaptic receptors and their associated proteins. Nonetheless, a nanomodule organization within the synapse in which these components are brought together to constitute an integrated subsynaptic component for vesicle release and transmitter reception is starting to emerge (Biederer et al., 2017). Data regarding the distance requirements in the nm scale for effective interaction between the VGCCs and the vesicle Ca^2+^ acceptor (Nakamura et al., 2015) as well as for the release and AMPA receptor locations (MacGillavry et al., 2013; Tang et al., 2016; Haas et al., 2018) has also indicated the importance of such close spatial organization. All in all, such a nanomodule should cover an area of no more than ~0.04 μm^2^ (Hruska et al., 2018), which is about the active zone areas of the smallest Schaffer collateral synapses onto CA1 pyramidal cells (CA3–CA1 synapses; Schikorski and Stevens, 1997). Interestingly, after chemically induced long-term potentiation (LTP), synapses acquire additional such nanomodules (Hruska et al., 2018), indicating that synaptic strength is a function of the number of such nanomodules acquired by a synapse (Lisman and Raghavachari, 2006).

Some time ago two of the authors of this article examined, using minimal stimulation and whole-cell recording, the release from single CA3–CA1 synapses in the neonatal rat (Hanse and Gustafsson, 2001a,b,c,d, 2002). These are small synapses likely to contain only one such nanomodule (Fiala et al., 1998). An analysis of the release pattern during brief train activation of these synapses strongly suggested univesicular release from a small population of vesicles, indicating that this putative nanomodule operates as a single functional release site. The vesicle pool was found to be dynamic in that the number of vesicles available for release at stimulation onset (referred to as the pre-primed pool) varied from trial to trial. Moreover, the release probability (P_r_) in response to the first action potential in the train (P_1_) varied much among these synapses, and depended, in addition to vesicle pool size, on a large diversity in vesicle release probability at the onset of stimulation (P_ves1_). This large diversity in P_ves1_ was instrumental in creating the large variation among the synapses in short-term plasticity behavior during train stimulation, from profound depression to large facilitation.

In this article we will describe in what manner and with which tools the experimental results from these neonatal synapses were acquired and processed, and discuss these results in the context of current understanding of the supramolecular structure of a nanomodule.

## Outline of a Nanomodule (The Functional Release Site)

The distribution of active zone and PSD areas of CA3–CA1 synapses in adult animals varies from ~0.01 to ~0.18 μm^2^, but is highly skewed with the vast majority of values between 0.02–0.04 μm^2^ (Schikorski and Stevens, 1997). Based on combined pre- and post-synaptic imaging (of vesicle and PSD proteins, respectively) it would appear that active zone areas below ~0.04 μm^2^ correspond to synapses containing a single nanomodule, and those with larger areas multiples of such nanomodules (Biederer et al., 2017; Hruska et al., 2018). With respect to the spatial organization of proteins on the postsynaptic side, the AMPA receptors are present throughout the PSD but are specifically clustered in a small area (hot spot, ~0.005 μm^2^) within the central region of the nanomodule (MacGillavry et al., 2013). On the presynaptic side, VGCCs, docked vesicles and release locations can also be found distributed throughout the active zone, but not randomly (Scimemi and Diamond, 2012; Nakamura et al., 2015; Tang et al., 2016; Éltes et al., 2017; Maschi and Klyachko, 2017). As recently proposed, the nucleus of a release location could be a nanoassembly of Munc13–1 (together with some other active zone proteins) that by contacting syntaxin-1 builds a docking/priming location for a single vesicle (Sakamoto et al., 2018). It is then assumed, albeit not demonstrated, that such a nanoassembly comes into close contact with a small cluster of VGCCs. Such a nanoassembly would have a diameter of 60–80 nm in total, that gives a nanoassembly area of 0.003–0.005 μm^2^, i.e., about one tenth of a nanomodule. Considering the number of docked vesicles that can be observed within an active zone area of <0.04 μm^2^ (Schikorski and Stevens, 1997) there should be some 2–6 Munc13–1 nanoassemblies in a small, 1-nanomodule, synapse. The vesicle-associated protein RIM1/2 also displays a clustered organization within the active zone (Tang et al., 2016), but with seemingly smaller number of hot spots than Munc-13 (Tang et al., 2016). A RIM1/2 nanoassembly is also organized in approximate register with the AMPA receptor hot spot, indicating a transverse nanocolumn for synaptic release/reception (Tang et al., 2016).

**Figure 1:**
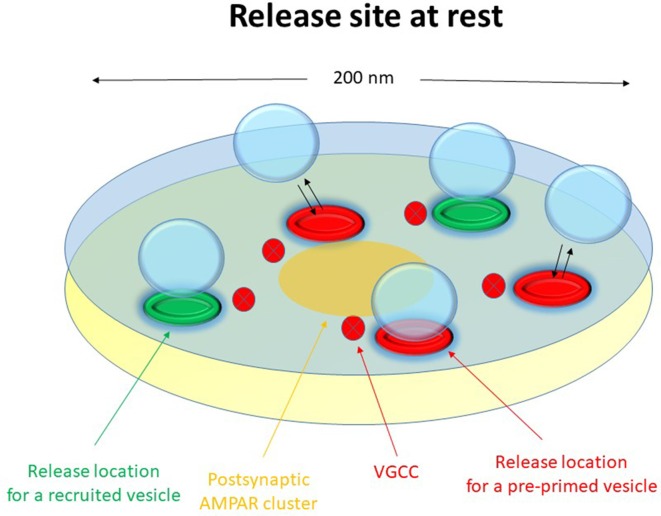
Schematic drawing of a functional release site (nanomodule) at rest. The schematic release site contains five release locations. The three red release locations constitute the pre-primed source pool, responsible for phasic release. Here one of these release locations has a docked and pre-primed vesicle. The two green release locations are for recruited vesicles, responsible for tonic release. Voltage-gated calcium channels are indicated in the presynaptic membrane and a nanocluster of AMPA receptors are indicated in the postsynaptic membrane.

The release machinery of a nanomodule might then consist of some 2–6 release locations (nanoassemblies) capable of binding a similar number of docked vesicles, distributed over an active zone area of about 0.02–0.04 μm^2^ but preferentially towards the center of the nanomodule opposite to the postsynaptic AMPA receptor hot spot. As indicated by the Munc13-1 vs. RIM1/2 discrepancy in number, there may be two functionally separate sets of release locations. A schematic drawing of such a nanomodule indicating these different sets of release locations (red vs. green) is shown in Figure 1.

## Minimal Stimulation Technique

**Figure 2:**
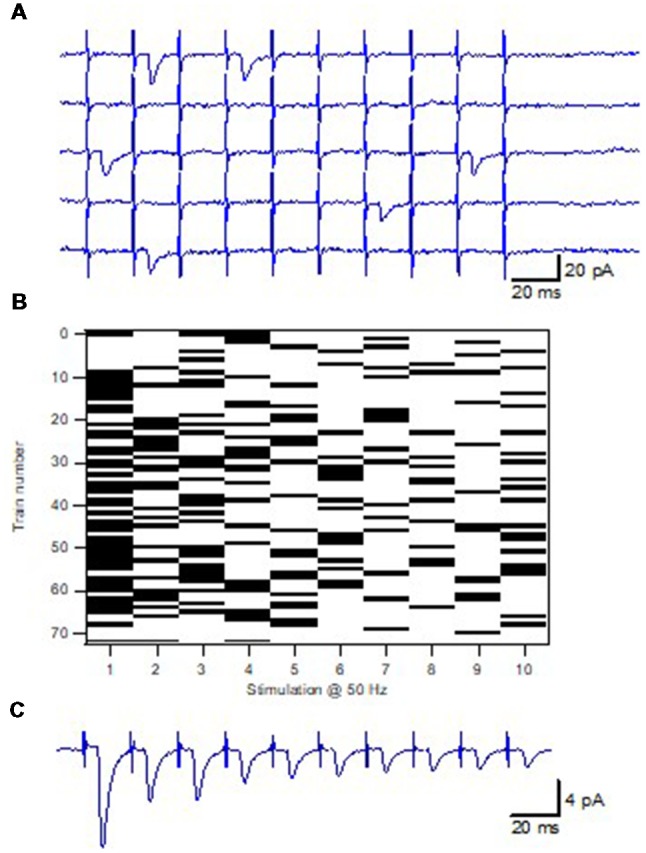
Minimal train stimulation of a unitary synaptic input.** (A)** Five consecutive example sweeps from one synaptic input in response to minimal train stimulation, 10 impulses 50 Hz. **(B)** Release pattern for the synaptic input shown in **(A)**. Release is indicated with a black bar and failure is indicated by a white bar. **(C)** Average train response for the synaptic input shown in **(A,B)**. Adapted from Hanse and Gustafsson (2001a).

In our study the minimal stimulation technique (Raastad et al., 1992; Stevens and Wang, 1995) was used to activate a single synapse onto a single CA1 pyramidal cell (Figure 2). This technique is possible to use for these neonatal synapses since there is good evidence electrophysiologically that the axons stimulated only have a single connection to a given postsynaptic cell (Hsia et al., 1998; Groc et al., 2002). Using brief train stimulation as test stimulation, axon activation and synaptic release goes hand in hand (in parallel), allowing for an unbiased selection of the CA3–CA1 synapses. Thus, the P_1_ values of the sampled synapses covered the full range of P_r_ values (in response to single action potentials) demonstrated for these synapses using population recordings (Wasling et al., 2004). In addition, the use of brief train stimulation as test stimulus also results in a sharp detection threshold for additional axon activations with variation in stimulation strength. The analysis of such single synapse activation experiments showed that the EPSCs, although varying substantially in amplitude, displayed a very narrow range of latency and time course (Hanse and Gustafsson, 2001c). If being an inclusion criterion such uniformity in EPSC time characteristics may cause a selection bias against synapses with multivesicular release. However, for our experiments this uniformity was a *post hoc* observation.

## Quantal Amplitude

**Figure 3:**
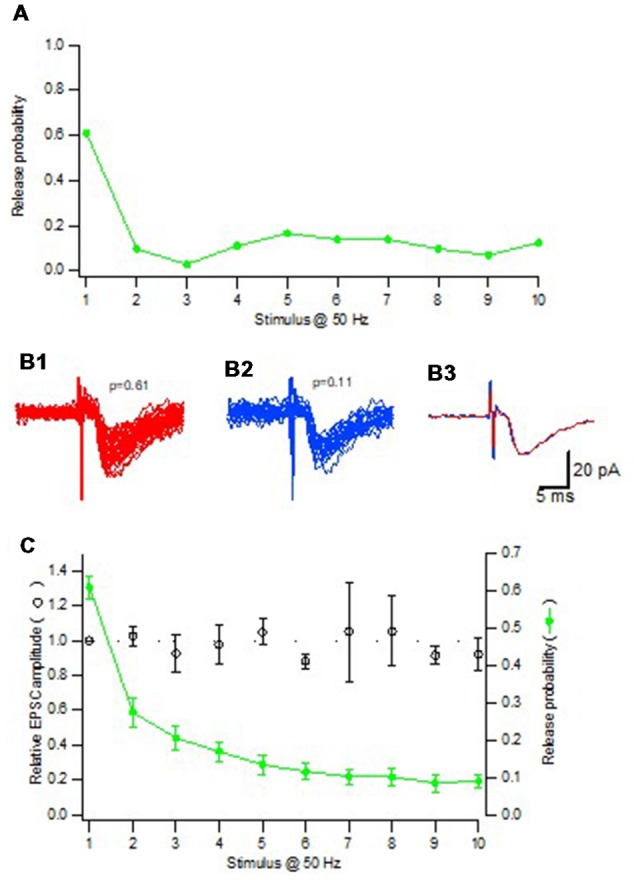
Quantal size is independent of release probability.** (A)** Release probability for one synaptic input repeatedly activated with a train consisting of 10 impulses at 50 Hz. **(B1)** All EPSCs (*n* = 44) from the 1st position in the train where the release probability was 0.61. **(B2)** All EPSCs (*n* = 22) from the 2nd to the 10th position in the train where the release probability was 0.11. To minimize the risk for potential influence of desensitization only EPSCs that were not immediately preceded by another EPSC were included. **(B3)** Average of the EPSCs included in **(B1,B2)** superimposed. **(C)** Summary plot (*n* = 19 synaptic inputs) shows both the relative amplitude of the EPSCs (open black circles) and the average release probability (filled green circles) as a function of stimulus position in the train. Adapted from Hanse and Gustafsson (2001c).

For any given synapse the evoked EPSCs varied substantially in amplitude, the coefficient of variation (CV) being mostly between 30%–60% among the synapses, associated with both normal and skewed distributions (Hanse and Gustafsson, 2001c). There was among the synapses no positive correlation between CV and EPSC amplitude (excluding failures), or release probability (P_r_), as might have been expected if multivesicular release contributes to the EPSC variation. To further examine whether multivesicular release contributes to the EPSC variation, the considerable change in P_r_ that can arise in a synapse during train activation was used. When selecting synapses with initial high P_r_ (>0.5) that displayed strong depression during the train stimulation, EPSC amplitude (excluding failures) was found to be independent of P_r_, thus seemingly excluding that the high P_r_ conditions result in multivesicular release (Figure 3). Such conclusion requires, however, that transmitter from a single vesicle does not come close to saturate the postsynaptic receptors. Experiments using cultured hippocampal neurons as well as 2nd postnatal week CA3–CA1 synapses in slice preparation have shown that the receptors are far from saturated, the median EPSC being <50% of the saturated response (Liu et al., 1999; McAllister and Stevens, 2000).

Given that the evoked EPSCs are generated from single vesicle releases, the variation in amplitude can result from the release of vesicles containing different amounts of transmitter either because of vesicle size variation (Sulzer and Edwards, 2000; Grabner and Moser, 2018) or of vesicle transmitter concentrations (Wu et al., 2007). In addition, vesicle release has been found to take place also outside the central region of the nanomodule where the AMPA receptors have their highest density (Maschi and Klyachko, 2017). Since a misalignment between release location and AMPA receptor hot spot of >100 nm can affect the EPSC amplitude (without obvious effects on the EPSC time course; Haas et al., 2018), such spatial mismatch may also contribute to the EPSC variability (Franks et al., 2003). However, creating such extra mismatch, using a truncated form of neuroligin that shifts the release locations away from the AMPA receptor hot spot, results in no more than ~20% decrease in quantal EPSC amplitude (Haas et al., 2018). Nonetheless, these results (Haas et al., 2018) suggest that, given a central position of the AMPA receptor hot spot within a nanomodule, the surface area of a nanomodule should not be >~0.04 μm^2^ for optimal activation of the AMPA receptors. This is in line with the observation of an increased number of nanomodules when synapses are strengthened after chemically induced LTP (Hruska et al., 2018). These results also suggest that our observation that the quantal EPSC is unaffected by its position in the train indicates that vesicles released initially vs. late in the train are released from locations ~equally close to the AMPA receptor hot spot.

## Pre-primed Pool

The P_r_ of a synapse is generally evaluated when activating the synapse at low frequencies, such as 0.2–0.033 Hz. The vesicle pool of interest for such release is the number of vesicles that are primed for release at just that instant of time when the action potential arrives, i.e., are pre-primed. The number of vesicles that can be released by prolonged stimulation of the synapses, or released by hypertonic treatment or other such means, may then not be very relevant. A common technique to evaluate this immediately releasable pool (but for a population of synapses) is to subject the synapses to a brief (10–20 impulses) high-frequency activation, and construct a cumulative synaptic response curve (Neher, 2015). After a few stimuli this curve becomes more or less linear, explained as the establishment of equilibrium between release and recruitment of vesicles. Extrapolation of this linear part to time zero then gives a measure of the vesicle pool available at stimulus onset. However, what is obtained is not the absolute pool size, but pool size expressed in units of the release probability. Moreover, the estimated value will depend on assumptions regarding when recruitment of new vesicles during the train stimulation actually begins. In addition, there is no way of knowing that this pool is fully depleted during the initial non-linear part of the cumulative curve.

### Determining the Pre-primed Pool

Brief train activation at high frequency was also used in our study to evoke release but the focus was on the interaction between release events occurring later in the train vs. that occurring to the 1st stimulus in the train (Hanse and Gustafsson, 2001d). Using that novel procedure, taking advantage of the variability in the number of release events in the various trials (Figure 2), it was first examined when during the stimulation train the occurrence of a 2nd release event is associated with a larger value of P_1_ (as compared to when only one release event had occurred). This analysis revealed that a 2nd release event that occurred within the first half of a 10-impulse train was associated with a larger P_1_, and was thus given by a pre-primed vesicle. Importantly, this vesicle added to P_1_ as if it acted independently of the vesicle that produced the 1st release event at that trial, and as if it had the same P_ves_ value. On the other hand, a 2nd release event that occurred in the second half of the train was not associated with a larger P_1_, and had thus been recruited to a primed state during the train. The pool of pre-primed vesicles, determining the value of P_1_, thus only constitutes a subpopulation of the vesicles released even during a 10-impulse 50 Hz stimulus.

**Figure 4:**
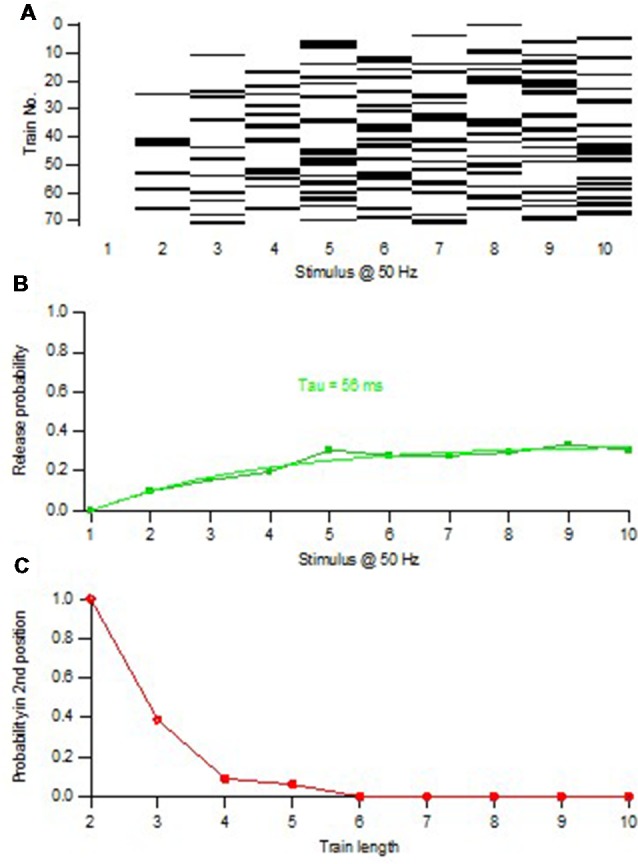
Minimal train stimulation of a synapse lacking pre-primed pool.** (A)** Release pattern for one synaptic input repeatedly activated with a train consisting of 10 impulses at 50 Hz. Release is indicated with a black bar and failure is indicated by a white bar. Note the absence of release in the 1st position of the train. **(B)** Release probability plotted against the position in the stimulus train. The release probability curve is fitted with exponential function indicating a time constant of increased release probability of 56 ms. **(C)** Release probability in the 2nd position of the train as a function of train length (increasing from 2 to 10). Only trials that up to the train length had contained one release event were selected for the calculation of the release probability in the 2nd position. Note that this curve decays to zero within five stimuli showing that no 1-release trials remain after the 5th stimulus. Adapted from Hanse and Gustafsson (2001d).

To estimate the size of this subpopulation, we adopted a variation of the above procedure to examine the timing of release events during the train. To explain this procedure one can consider a single release location that acquires and releases a single vesicle (with a certain probability) one at a time at a certain rate, and expose it to repeated trials of train stimulation. Thereafter the relation between P_1_ and train length is examined, selecting only trials in which one release event occurs (1-release trials). With a train length of 1, P_1_ will of course be 1. With increasing train length, trials with the single vesicle released at later positions in the train will occur, and P_1_ will successively decrease. Moreover, since the release occurs at a certain rate, trials in which the vesicle was released in position 1 tend to be the first to display a 2nd release event, and no longer be counted as 1-release trials. These trials will be 2-release trials, removing them (when a vesicle is released in position 1) from the calculation of P_1_. Thus, the P_1_-train length curve will continually decay and reach zero after a time reflecting the recruitment rate. Such P_1_-train length relations were indeed also found when examining synapses lacking initial release (Figure 4), or when starting the analysis after the depletion of the pre-primed pool (Hanse and Gustafsson, 2001d).

**Figure 5:**
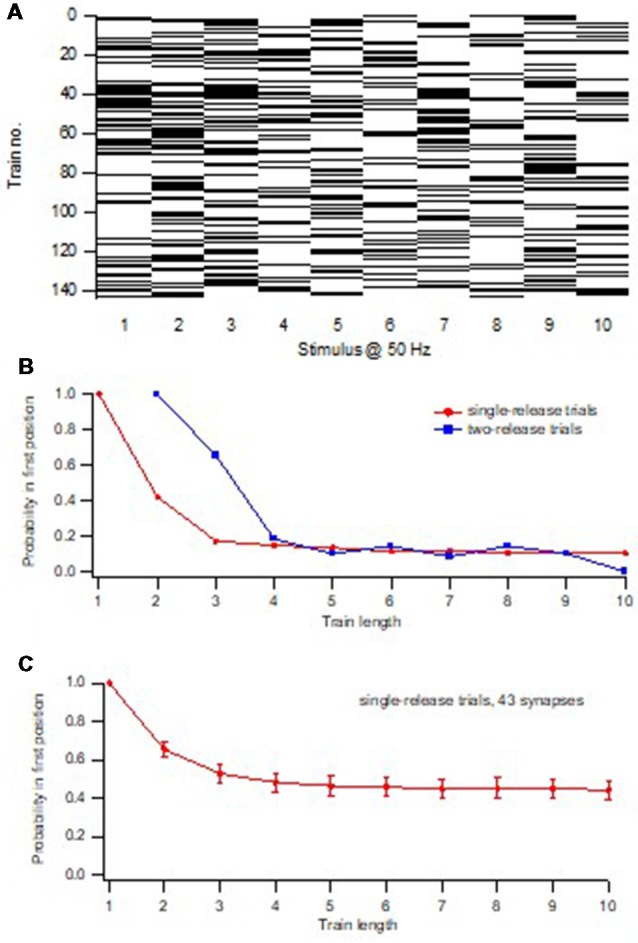
Determination of the pre-primed pool and P_ves1_.** (A)** Release pattern for one synaptic input repeatedly activated with a train consisting of 10 impulses at 50 Hz. Release is indicated with a black bar and failure is indicated by a white bar. **(B)** Release probability in 1st position of the train as a function of train length (increasing from 1 to 10). Red squares represent trials that up to the train length had only contained one release event, and those trials were selected for the calculation of the release probability in the 1st position. In other words, for each train-length x, all trials that contained a single release event in the first × positions were selected out from all trials in a given experiment, and P_1_ was calculated. Note that this curve decays to a plateau within five stimuli indicating that no 1-release trials remain after the 5th stimulus. Blue squares represent trials that up to the train length had contained two release events, the second release event in the last position of the examined train length. **(C)** Summary graph of single release trials from 43 synaptic inputs. Adapted from Hanse and Gustafsson (2001d).

Consider instead a release location that contains a single vesicle at the onset of stimulation, and onto which there is no new recruitment. The P_1_-train length curve, selecting only 1-release trials, will initially look the same as in the above example. However, at a train length corresponding to the maximum number of stimuli needed to release that vesicle, the curve will flatten out and reach a plateau level at a P_1_ value that is equal to P_ves1_. The reason for this plateau is that all trials will remain 1-release trials when train length becomes longer because there is no further release (due to lack of recruitment). If now recruitment is added to this release such that, as in the above scenario, trials in which the vesicle was released in position 1 will be the first to display a 2nd release event, the plateau will disappear and the curve decay to zero. If, on the other hand, the recruited vesicles are recruited/released at random with respect to the release of the 1st vesicle, 1-release trials used for the calculation of P_1_ will disappear from the analysis regardless of the position in the train at which the 1st vesicle was released. The curve will thus flatten out and reach a plateau level at a P_1_ value that is equal to P_ves1_. Such P_1_-train length relations with a plateau were indeed also invariably found when the synapses were examined starting from the 1st stimulus position in the train (Figures 5B,C).

The existence of such a plateau thus suggests that the pre-primed pool is a subpool separate from the recruited pool of vesicles, and that it should roughly correspond to the number of vesicles released before the plateau is reached. To further sharpen when during the train the pre-primed pool is used up, and the recruited pool has taken over, for each synapse the P_1_-train length curve was also determined for 2-release trials. From the intersection of this curve with that obtained using 1-release trials, the position in the train at which a 2nd release event no longer affected P_1_, i.e., no longer came from the pre-primed pool, could be determined (Figure 5B). The average pre-primed pool for a synapse was thereafter estimated as the cumulative release occurring prior to that position. Likewise, the pre-primed pool at each individual trial for a synapse was estimated as the number of release events in that trial occurring prior to that position (Hanse and Gustafsson, 2001b).

### Trial-to-Trial Variation in Pre-primed Pool Size, and Pool Size Distribution

For any given synapse, the pre-primed pool was found to vary in size from trial to trial, mostly between zero and three. Thus, at some trials pre-primed vesicles were completely absent, the fraction of such trials varying between 10% and 50% among the synapses (see Figure 5A). Such a stochastic trial-to-trial variation in the number of vesicles released has also been noted by others (Trigo et al., 2012). To obtain a measure of the form of the pool size distribution, values from synapses of about equal average pre-primed pool size were compiled (Hanse and Gustafsson, 2001b). This procedure resulted in distributions that agreed with binomial ones with a probability of 0.3 of the primed state, independent of pre-primed pool size. This would suggest that the pre-primed pool is part of a three times larger pool (the pre-primed source pool) that in a dynamic fashion shapes the number of vesicles primed at stimulus onset. This partial priming of the pool is not a consequence of a very slow priming rate. In some experiments the trains were also evoked once every 30 s (instead of every 5 s; Hanse and Gustafsson, 2001d). This slowing of repetition rate increased the pre-primed pool by only 15%, demonstrating that even at such slow stimulus rates most vesicles in the pool are not pre-primed at the arrival of an action potential. On the other hand, it also shows that the pre-priming rate is nevertheless quite slow, taking more than 5 s.

The P_ves1_ value for a synapse could thus be obtained from the P_1_ value when only 1-release trials were used, and was found to vary among the synapses from <0.1 to almost 1.0, on average 0.43. Together with an average pre-primed pool of close to 1.0 (see below), the average P_1_ should be ~0.4, which agrees well with the average P_1_ value of 0.42 for our synapse population (excluding synapses lacking initial release; Hanse and Gustafsson, 2001d). To examine this issue also for the individual synapses, the estimated average pre-primed pool and P_ves1_ values were obtained for each synapse from the subgroups of 1- and 2-release trials, respectively. The pre-primed vesicles were then allowed to operate independently to cause the release of a single vesicle, according to the equation P_1_ = 1 – (1 − *P*_ves_)^pool^. The P_1_ values calculated from these values of pre-primed pool and P_ves1_ were found to agree well with the experimentally obtained P_1_ values observed using all the trials (Hanse and Gustafsson, 2001d).

### Release Dependence Within a Paired Stimulus; Effect of a Dynamic Pre-primed Pool

The (average) pre-primed pool sizes estimated in the above manner varied among the synapses from 0.5 to 2.0 with a skew towards lower values, the average value among the synapses being 1.03 (Hanse and Gustafsson, 2001d). Such small pool values beg the question of how to explain the well-known fact for CA3–CA1 synapses that, using paired-pulse activation, the P_r_ to the 2nd stimulus (P_2_) is the same whether or not there is release to the 1st stimulus (P_2 release_/P_2 failure_ ≈ 1; Stevens and Wang, 1994; Isaac et al., 1996; Hjelmstad et al., 1997; Hanse and Gustafsson, 2001c). Certainly, with such a small pool the release of 1 vesicle by the 1st action potential would be expected to affect P_2_. However, simulating such release indicated, on average, little release dependence (Hanse and Gustafsson, 2002). This is because with a binomially distributed trial-to-trial variation in pool size, release in response to the 1st stimulus will preferentially occur on those trials in which more vesicles are primed, and* vice versa*. The number of pre-primed vesicles remaining for the 2nd stimulus can then be equal independent of whether release occurred, or not, in response to the 1st stimulus. Proper consideration of such a mechanism for release success or failure can be relevant for the interpretation of causes of paired-pulse plasticity (see “Discussion” section in Hanse and Gustafsson, 2002). Simulation also showed some deviation from a ratio of 1 depending on the value of P_1_, the ratio being somewhat <1 at low P_1_ and >1 at high P_1_. Such deviation, which does not occur if multivesicular release is allowed in the simulations, was also observed for the experimentally observed values (Hanse and Gustafsson, 2002). It should be noted that a consequence of the dynamic pool is thus that the pre-primed pool size will actually appear to be reduced (“depleted”) independently of whether release occurred, or not.

As will be discussed later in more detail (see “Multivesicular Release” section), other authors see the nanoassembly rather than the nanomodule as the quantal release site, implying several independent release sites within a nanomodule (Sakamoto et al., 2018). Since the number of release sites was found to be equal to the number of vesicles in the readily releasable pool, each of these release sites should at rest be fully occupied with a single vesicle. Following the release from such a site, this site will be replenished with a new vesicle which will subsequently be released. Such a release scenario is not consistent with our data. Thus, the P_1_-train length relation will not display any plateau but decays to zero because of the cyclical manner of release (Figure 4). Moreover, release to the 1st stimulus will always be associated with a smaller P_2_, i.e., (P_2 release_/P_2 failure_ < 1). For example, in the case of a synapse with only a single release site, release to the 1st stimulus will always result in zero release to the 2nd stimulus.

## P_ves1_,P_ves_ Normalization and Paired-Pulse Plasticity

CA3–CA1 synapses activated at low frequency by single action potentials or by brief trains are very heterogeneous with respect to P_r_ and P_1_, respectively, also in the neonatal rat. Note that while P_r_ and P_1_ values both refer to release probabilities obtained in response to the 1st action potential following a period of rest, they may not be the exactly the same because of lingering effects of short-term plasticity. Nonetheless, both these release probabilities vary among the synapses from well below 0.1 to close to 1 with a distribution skewed towards the lower values (Dobrunz and Stevens, 1997; Hanse and Gustafsson, 2001d; Wasling et al., 2004). While the variation in P_r_ has generally been attributed to a variation in pool size among the synapses (Dobrunz and Stevens, 1997), our data suggested that a variation in P_ves1_ (<0.1–0.9) was an even more important factor for the P_1_ heterogeneity. This dependence of P_1_ on P_ves1_ is not in contrast to the previous notion of a dependence on pool size since no correlation was found between the size of the pre-primed pool and P_ves1_ (Hanse and Gustafsson, 2001d). Thus, the variation in P_ves1_ will not actually alter the overall effect of a variation in pool size on P_1_.

**Figure 6:**
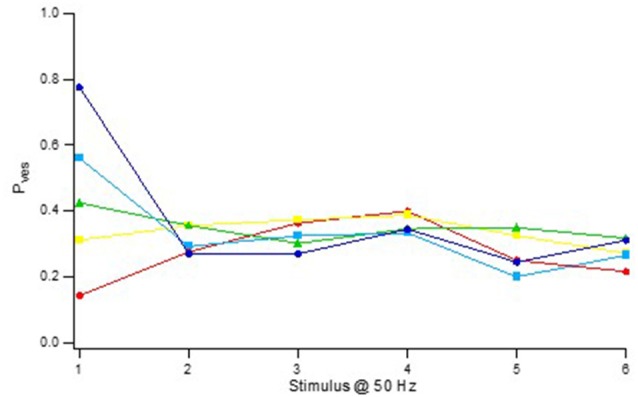
Activity-dependent normalization of P_ves_. Vesicle release probability (P_ves_) as a function of stimulus position in a 50 Hz train. The synaptic inputs were divided into five groups according their P_ves_ in the 1st stimulus position. P_ves_ was calculated using the equation *P*_ves(n)_ = 1 – (1 − *P*_(n)_)^1/pool(n)^, where P_(n)_ is the release probability at stimulus position *n* and pool_*(n)*_ is the size of the pre-primed pool at the nth stimulation. The pre-primed pool was estimated after subtraction of the average release probability curve for synapses lacking pre-primed pool (“Zero P_1_” in Figure 8C). Adapted from Hanse and Gustafsson (2001a).

Notably, this P_ves_ heterogeneity was only true with respect to the 1st stimulus in the train (P_ves1_), the P_ves_ values computed for releases to the 2nd stimulus (P_ves2_) displaying a much more narrow distribution (0.2–0.4; Figure 6) with no correlation between P_ves1_ and P_ves2_ (Hanse and Gustafsson, 2001a). For any given synapse the P_ves_ value for later releases from the pre-primed pool stayed at the level of P_ves2_. This P_ves_ normalization did not require release but was secondary to the action potential itself and/or its associated calcium influx.

Thus, during synaptic activity the P_ves_ heterogeneity among the synapses largely disappears, thereafter to become re-established by synaptic inactivity. This establishment of P_ves1_ heterogeneity by synaptic inactivity and its removal by activity makes many of the CA3–CA1 synapses rather unresponsive to sporadic arrivals of action potentials but more responsive after their arrivals, while other synapses are made very responsive to such arrivals but much less so thereafter. Since the P_ves_ normalization does not require release, high P_ves1_ and low P_ves1_ synapses are thus subjected to a release-independent depression and facilitation, respectively. The inactivity-induced establishment of P_ves1_ heterogeneity followed by a release-independent P_ves_ normalization is thus instrumental in producing the heterogeneity in facilitation/depression behavior among the synapses appearing during the first few stimuli (such as paired-pulse plasticity), and can be seen as a means to create differential dynamics within a synapse population.

A similar dissociation between a large heterogeneity in initial release and a more narrowly distributed later release has also been described in another well-studied synapse, the Calyx of Held synapse (Taschenberger et al., 2016). This behavior was interpreted by these authors as the presence in some proportion of the synapses of superprimed (high P_ves_) vesicles. These vesicles will result in an initial high P_r_, but they are rapidly used up, allowing vesicles with normal P_ves_ to decide later release. This interpretation does not agree with ours since what appears to be “superprimed” in the high P_r_ CA3–CA1 synapses is not the vesicle but the release location. Moreover, there should then not only be “superprimed” but also “subprimed” locations, creating high and low P_r_ synapses, respectively. Furthermore, while the superprimed state in Calyx of Held synapses is thought to disappear in a release-dependent fashion (depletion of the superprimed vesicles), the P_ves_ normalization occurs in a release-independent manner.

We have no explanation for the large P_ves1_ heterogeneity among the neonatal CA3–CA1 synapses. One factor that controls P_ves_ is the density of VGCCs contributing to the trigger calcium (Éltes et al., 2017). However, since a single action potential can switch P_ves1_ to a new value (P_ves2_) that is completely unrelated to P_ves1_, such a quantitative difference in VGCCs does not seem likely. Another important factor in deciding P_ves_ is the distance between the VGCCs and the vesicle calcium acceptor synaptotagmin-1. Should this distance exceed 100 nm, P_ves_ would be reduced to negligible levels (Nakamura et al., 2018). One may then speculate that during rest some synapses keep their docked/primed vesicles more distant from the VGCCs, this difference nullified by action potential-induced calcium entry. Finally, other important regulators of P_ves_ are the vesicle-related proteins Munc13–1 and Munc18 (Lai et al., 2017) and synaptotagmin-7 (Jackman et al., 2016). Since these proteins affect the energy barrier for fusion and can bind calcium, one can also envisage activity-dependent changes in their influence on P_ves_.

## Multivesicular Release

Our analysis suggests that a single nanomodule, despite containing a number of docked vesicles and release locations, functions as a single release site releasing at most a single vesicle at the arrival of an action potential. As demonstrated a long time ago, release from a CA3–CA1 synapse is followed by a few ms of release refractoriness that may explain such univesicular release from a population of vesicles (Stevens and Wang, 1995; Dobrunz et al., 1997; Hjelmstad et al., 1997). While there is no existing explanation for such refractoriness, there are several manners in which such lateral inhibition of release following the exocytosis of one of the vesicles could occur (Nadkarni et al., 2010). Nonetheless, evidence for multivesicular release has been presented for a number of synapses, including the CA3–CA1 synapses (Oertner et al., 2002; Christie and Jahr, 2006; Ricci-Tersenghi et al., 2006), and the notion that each docking site is an independent release site is now considered the favored one (Rudolph et al., 2015; Pulido and Marty, 2017). However, even quite small synapses (active zone areas of 0.05–0.1 μm^2^) may contain more than one nanomodule (Hruska et al., 2018), resulting in multivesicular release but from morphologically separate release regions (nanomodules) within an active zone, assuming that a possible lateral inhibition of release among the vesicles is restricted to vesicles within a nanomodule. On the other hand, should multivesicular release occur from a single nanomodule, there should be no lateral inhibition, and thus no release refractoriness. If such lack of refractoriness exists in such synapses remains to be demonstrated (Nadkarni et al., 2010). In addition, the time resolution in the method used to detect release must also be such that a 2nd release event is not explained by asynchronous release.

Nonetheless, a favored notion today is that release from an active zone is multivesicular (Rudolph et al., 2015; Pulido and Marty, 2017), and, importantly, that each docking site works as an independent release site. Recent evidence for this notion can be found in the study by Sakamoto et al. (2018) which combined examination of the release from individual hippocampal synapses using a glutamate imaging technique with studies of the nanoscale supramolecular organization of the active zone protein Munc13–1, thought to be important for vesicle priming. To estimate the number of independent release sites for the synapse examined, the authors used the multiple probability fluctuation analysis in which the variance of the synaptic response is estimated at various values of P_1_ (Saviane and Silver, 2007) obtained e.g., by varying the extracellular Ca^2+^/Mg^2+^ ratio. For the synapse population examined this estimated number of release sites was found to be correlated in an 1:1 relation with the number of Munc13–1 nanoassemblies, suggesting that a single such nanoassembly operates (together with some other active zone proteins) as an independent release site. Also the readily releasable pool of vesicles for each synapse was estimated, using the cumulative synaptic response curve given by brief high-frequency stimulation. This pool was also found to be correlated in a 1:1 relation to the number of release sites, indicating that, at rest, each release site (nanoassembly) is occupied by one vesicle each. These vesicles would then constitute a readily releasable pool of vesicles that is depleted within a few high frequency stimuli. Release thereafter would come from the fast replenishment of these same release sites with vesicles docked/primed following the onset of stimulation.

While these results clearly appear to favor a release behavior quite distinct from that favored by our results, there are certain aspects that have to be considered. As noted above (“Release Dependence Within a Paired Stimulus; Effect of a Dynamic Pre-primed Pool” section), our results indicated a pre-primed pool that on average was close to 1 among the synapses. Thus, also a synapse that contains several nanomodules within an active zone, each nanomodule consisting of several nanoassemblies, would also on the average have an equal number of release sites and of readily releasable vesicles. In the Sakamoto et al.’s (2018) article there is no mention of either the active zone areas or the number of docked vesicles within these areas. Nonetheless, from their published records (see their Figure 4C), the active zones were at least 2–5 times larger than the 0.04 μm^2^ area taken by us as the upper limit of a nanomodule. Thus, active zones of this size could at least explain the number of release sites (1–6) in the form of nanomodules found in the vast majority of the synapses examined by these authors.

The other aspect to be considered is the equal number of nanoassemblies and release sites reported in this article (Sakamoto et al., 2018), each nanoassembly believed to be occupied at rest by a docked/primed vesicle and serving as the quantal release site. However, no independent evidence for such a match between vesicle and nanoassembly number was provided. In fact, estimates of docked vesicle number in cultured hippocampal synapses (Schikorski and Stevens, 1997) would suggest a considerably higher number of such vesicles (for active zone areas comparable to those indicated in Figure 4C of Sakamoto et al., 2018) than the number of nanoassemblies reported by Sakamoto et al. (2018). The variation in size among the observed nanoassemblies (Figure 4C of Sakamoto et al., 2018) also begs the question of whether all of these represent discrete entities. In fact, in a similar recent study, using the *Drosophila* neuromuscular junction, release sites corresponding to a nanomodule in size appeared to contain several such assemblies (Reddy-Alla et al., 2017). Thus, while Sakamoto et al. (2018) make a rather strong case for multiple release sites within an active zone, they do not necessarily set aside our notion that a nanomodule, containing a number of docked vesicles and release locations, serves as the quantal release site.

Another form of experimental approach to demonstrate multivesicular release is the use of a weak AMPA receptor antagonist. Thus, when multivesicular release occurs, the glutamate concentration in the synaptic cleft will be higher and the weak antagonist will have less effect on the synaptic response. Using this technique onto third week hippocampal synapses, synaptic field responses evoked under conditions of high release probability (to increase the likelihood of multivesicular release) were found to be significantly less affected by such a receptor antagonist than responses observed under control conditions (Christie and Jahr, 2006). While such a result strongly suggests multivesicular release at those synapses, it does not necessarily invalidate our notion of nanomodule univesicular release. Postsynaptic subdomains of nanomodule dimensions, based on PSD-95, can be separated by less than about 200 nm (Fukata et al., 2013), and simulations have indicated that AMPA receptors can be activated from release locations several 100 nm away (Haas et al., 2018). Thus, one cannot exclude that glutamate released from one nanomodule may contribute to AMPA receptor activation at an adjacent nanomodule. Thus, we believe that a proper interpretation of studies using weak receptor antagonist has to await more knowledge regarding a possible cross-talk among the nanomodules within an active zone.

## Initial vs. Late Release and Vesicle Recruitment

**Figure 7:**
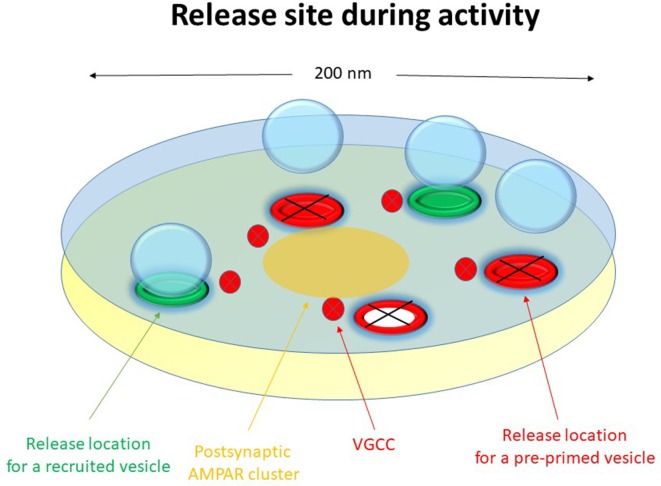
Schematic drawing of a functional release site (nanomodule) during activity. The schematic release site contains five release locations. The three red release locations constitute the pre-primed source pool, responsible for phasic release. The two green release locations are for recruited vesicles, responsible for tonic release. During activity, priming and release occur at the release locations for recruited vesicles and the release locations for pre-primed vesicles do not contribute as indicated by black crosses. The red and white release location indicates recent exocytosis of a pre-primed vesicle. Voltage-gated calcium channels are indicated in the presynaptic membrane and a nanocluster of AMPA receptors are indicated in the postsynaptic membrane.

Our data suggest that the neonatal CA3–CA1 synapses have a pre-primed source pool that varies among the synapses from two to six vesicles, and which is responsible for the pre-primed pool (Hanse and Gustafsson, 2001d). The size of this source pool is thus in rough agreement with the number of docked vesicles and release locations in a nanomodule. The pre-primed pool averages one-third of the pre-primed source pool, but varies from trial to trial mostly from zero up to three vesicles (Hanse and Gustafsson, 2001b). During brief train stimulation only the pre-primed vesicles of that source pool can participate in the release (Figure 1). This is because, as indicated by the plateau phase of the P_1_-train length curve, the vesicles released later in the train are released in a random manner with respect to those released from the pre-primed pool. They must therefore come from a separate pool of vesicles that are released at a separate set of release locations than those used by the pre-primed vesicles (Figure 7, green release locations). It may then be envisaged that the priming of the pre-primed source pool of vesicles (that occurs during rest and is slow) takes place at the actual release location and that the vesicles that are not in a primed state at stimulation onset hinder further release from these locations (release locations with black crosses in Figure 7). The vesicles released later in the train have then been recruited and primed (“post-primed”) in an activity—dependent manner with a fast priming rate to the other set of locations (Figure 7, green release locations). If these recruited vesicles are docked prior to their priming, they would add a few additional docked vesicles/release locations to the 2–6 vesicles constituting the pre-primed source pool. It is tempting to associate the pre-primed source pool to the possible subgroup of release locations containing RIM1/2 nanoassemblies (Tang et al., 2016). Some support for this notion comes from experiments using RIM1α knock-outs in which the later release is left unaffected while the initial release is reduced (Calakos et al., 2004).

This distinction between a pre-primed source pool and a recruited pool deduced from the P_1_-train length curves is also supported by the release behavior of some synapses that only show release to the first 2–3 stimuli of the train as well as of some synapses displaying no release until the 2nd or 3rd stimulus (Hanse and Gustafsson, 2001d), indicating an absence of a recruited and a pre-primed pool, respectively. Also synapses with a dip in the release at the 3rd–4th stimulus positions, indicating a temporal separation between the releases from two distinct pools, were observed (Hanse and Gustafsson, 2001d). A further test of this two pool idea would have been to examine for each trial the correlation between release events belonging to the pre-primed and the recruited pool, respectively. That is, if the pools are distinct, the number of recruited release events should be the same whether a trial shows 2–3 pre-primed release events or no such events. Unfortunately, no such analysis was thought of at the time of the publication of our studies.

Another manner to demonstrate a difference between the pools would be to condition a 10-impulse train by either a brief train (such as a 3-impulse train that would release predominantly from the pre-primed pool) or by a 10-impulse tetanus producing release from both pools. In fact, such experiments have been done, showing that the brief train only reduces the initial part of the evoked response while the longer train affects also the later release (Andersson and Hanse, 2011).

### Two Parallel Vesicle Pools in a Nanomodule for Initial (Phasic) and Later (Tonic) Release, Respectively

From the above it appears that the vesicles within a nanomodule can interact with two separate sets of release locations (Figure 1), possibly differing with respect to the type of molecules that constitutes the release location (see above). At one of these sets (Figure 7, green release locations), vesicles are released in a cyclical fashion, and the vesicles interacting with these release locations produce the later steady state, or “tonic,” release during the stimulation train. These vesicles should not be able to become pre-primed, i.e., to be primed during inactivity. Instead they will become primed within 50–100 ms after onset of activity (post-primed) likely as a consequence of increased cytoplasmic Ca^2+^. Whether these vesicles are docked prior to the onset of activity cannot be decided from our data. The P_ves_ of these vesicles is also unknown. While the P_ves_ of vesicles released from the pre-primed pool can be estimated to be ~0.2–0.4 for stimulus positions beyond the first, it cannot be directly assumed that such P_ves_ values also hold true for this recruited pool of vesicles. However, considering that these vesicles are released in a cyclical manner, the main determinant of P_r_ in this part of the train would not be P_ves_, but rather the recruitment to the release location, and the number of such locations within a nanomodule. At the other set of release locations (Figure 7, red release locations) the vesicles can become docked/primed during inactivity, i.e., become pre-primed, and following the onset of activity a given release location can only, at most, release one vesicle. Generally, only a subset of the vesicles in this pool is in a primed condition at the onset of activity, and the size of this subset varies from trial to trial. The vesicles interacting with these release locations thus produce the very initial (“phasic”) release at the onset of activity.

When considering these vesicle pools to be in parallel, we do not suggest that vesicles in these two pools necessarily differ from each other. Instead, any given vesicle may enter either of these pools. It is not until it interacts with a release location that it enters into one of these pools. Thus, what may operate independently of (or parallel to) each other are the two sets of release locations, “phasic” and “tonic” ones, respectively. If such independence would be true, release from these locations would not only appear in isolation, as can be observed in some synapses, but also proceed in an additive manner when release from these locations may overlap temporally. That this may be the case is suggested by using the (average) release from synapses not displaying any initial release as a template for release from the “tonic” pool. Thus, subtraction of this template from the total release in synapses exhibiting initial release results in pre-primed pool values in good agreement with those obtained with the procedure described earlier (Figure 5; Hanse and Gustafsson, 2001d). On the other hand, as described earlier (“Determining the Pre-primed Pool” section), we observed that a 2nd release event that occurred within the first half of a 10-impulse train was associated with a P_1_ value corresponding to that expected from two pre-primed vesicles. This result would suggest that comparably few 2nd release events in the first half of the train came from the recruited pool, indicating a bias against the release of post-primed vesicles prior to the release from the pre-primed pool. However, we cannot exclude that this observation simply reflects the fact that the total release during a 10-impulse 50 Hz train is predominantly from the pre-primed pool (60%; Hanse and Gustafsson, 2001d), and that the 1st release from the recruited pool also for temporal reasons is more likely to be a 3rd than a 2nd release event.

### Short-Term Synaptic Plasticity and Shift in Release Location

**Figure 8:**
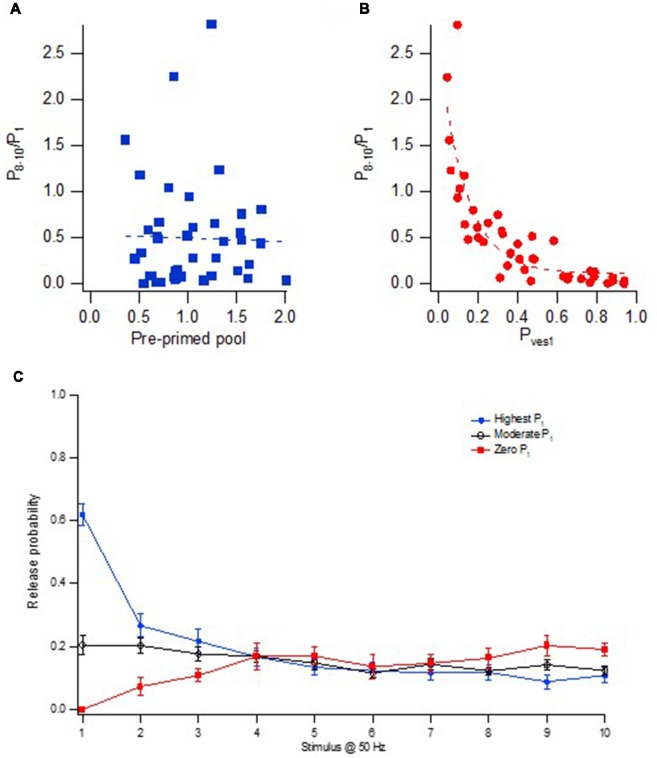
Heterogeneity in frequency facilitation/depression among the synapses.** (A)** Relationship between facilitation/depression (P_8–10_/P_1_) and the size of the pre-primed pool for 43 synaptic inputs. **(B)** Relationship between facilitation/depression (P_8–10_/P_1_) and P_ves1_ for 43 synaptic inputs. **(C)** Release probability at each stimulus position in a 50 Hz train for three groups of synaptic inputs; High P_1_ (*n* = 21, blue circles), moderate P_1_ (*n* = 22, black open circles) and synaptic inputs with zero P_1_ (*n* = 9, red squares). Adapted from Hanse and Gustafsson (2001a).

The short-term plasticity during brief train activation was in our studies quantified as the P_8–10_/P_1_ ratio, i.e., the ratio between the average release probability at stimulus position 8–10, and the release probability at the 1st stimulus position. This form of short-term plasticity (frequency facilitation/depression) was well correlated with P_ves1_ but not with pre-primed pool size (Figure 8). Important in shaping this short-term plasticity would be the form of correlations that exists between the factors that decide release from the “phasic” and “tonic” pools, respectively. Within a nanomodule there is a strong correlation between the number of docked vesicles and the nanomodule area, as judged from the data from more adult CA3–CA1 synapses (Schikorski and Stevens, 1997). If so, the variation in pre-primed pool size among the synapses is explained by variation in nanomodule area, and, likewise, also implies a correlated variation in the number of “tonic” release locations. Since both the “phasic” and “tonic” release then will co-vary with a variation in nanomodule size, this short-term plasticity will not depend on pre-primed pool size (Figure 8A) and thus not on nanomodule size. Instead, the variation in short-term plasticity among the synapses will be shaped by the correlation between P_ves1_ and recruitment per “tonic” release location. As a measure of recruitment per release location we used the P_8–10_ value divided by the pre-primed pool size (as an indicator of nanomodule area). Interestingly, these two parameters were found to be strongly negatively correlated among the synapses (Hanse and Gustafsson, 2001a), together producing the very large variation in facilitation/depression behavior among the synapses shown in Figures 8A,B.

In Figure 8C is plotted the release probability curves for three groups of synapses, those with high initial release probability (P_1_), those with moderate initial release, and those without initial release, these groups of synapses exhibiting essentially the same absolute amount of late release. That is, on the average among the synapses, the late P_r_ is independent of the initial release. For the synapses exhibiting initial release, this independence from initial release is likely explained by the negative correlation between P_ves1_ and recruitment per “tonic” release location. Thus, the influence of a larger nanomodule area (and more release locations) on both P_1_ and P_8–10_ will in itself result in both a higher late P_r_ and a higher P_1_. However, synapses with high P_ves1_ will not only contribute to a high P_1_ but also to a small late P_r_ (because of a low recruitment per release location), offsetting the effect of more “tonic” release locations in high P_1_ synapses.

When it comes to the short-term synaptic plasticity that will be present when stimulation frequency is altered from the 50 Hz used in our study to lower frequencies such as 1 Hz and beyond, it becomes more difficult to delineate the manner in which the two sets of release locations will participate in release. This is because we still know too little about the kinetics of several of the involved processes, such as the reestablishment of P_ves1_ and of the pre-primed pool during inactivity. For example, if the P_ves_ heterogeneity should become re-established in parallel with the recovery of the pre-primed pool (>5 s), one might expect to see a quite prolonged paired-pulse depression as well as a frequency depression in the <1 Hz frequency range. Thus, with increased stimulation frequency and number of stimuli, release will increasingly shift from the “phasic” to the “tonic” pool. In fact, when studying synapses onto the distal dendritic tree in stratum lacunosum-moleculare (SLM) of the neonatal CA1 neurons (SLM—CA1 synapses), these synapses display such a depression (Ma et al., 2016), indicating that such a shift may occur. On the other hand, this form of plasticity was not observed for the synapses (neonatal CA3–CA1 synapses; Ma et al., 2016) from which our data are taken. A possible explanation could be that the manifestation of this plasticity depends upon the net effect on release of the P_ves_ normalization seen over a population of synapses. Thus, if the P_ves_ normalization results in a P_ves2_ that on average is greater than the average P_ves1_ for a synapse population, this will mask the depression. Further studies will obviously be needed to understand whether the depression observed in the SLM–CA1 synapses is actually explained by such a shift in release location and, if so, if the above explanation for its absence in the CA3–CA1 synapses holds true.

It can finally be noted that this concept of two separate sets of release locations for initial and later release is not a new one. In recent times, this concept has been suggested for neuromuscular synapses in zebra fish, although in this case a location within separate release sites rather than within a single release site was the preferred interpretation (Wen et al., 2016). Also, although discussed in terms of vesicles rather than of release locations, the parallel model with two populations involved in initial and later release, respectively, at the Calyx of Held synapses (Mahfooz et al., 2016; Taschenberger et al., 2016), quite resembles the release mechanism described here for the hippocampal synapses.

## Author Contributions

All authors took part in designing and writing the manuscript.

## Conflict of Interest Statement

The authors declare that the research was conducted in the absence of any commercial or financial relationships that could be construed as a potential conflict of interest.
